# Epigenetic regulation of human-specific gene expression in the prefrontal cortex

**DOI:** 10.1186/s12915-023-01612-3

**Published:** 2023-05-24

**Authors:** Weifen Sun, Gangcai Xie, Xi Jiang, Philipp Khaitovich, Dingding Han, Xiling Liu

**Affiliations:** 1 Shanghai Key Laboratory of Forensic Medicine, Shanghai Forensic Service Platform, Academy of Forensic Science, Ministry of Justice, Shanghai, 200063 China; 2 CAS Key Laboratory of Computational Biology, CAS-MPG Partner Institute for Computational Biology, Shanghai Institute of Nutrition and Health, CAS, Shanghai, 200031 China; 3 Shanghai Institute of Hematology, State Key Laboratory of Medical Genomics, National Research Center for Translational Medicine at Shanghai, Ruijin Hospital, Shanghai Jiao Tong University School of Medicine, Shanghai, 200025 China; 4 Skolkovo Institute of Science and Technology, Moscow, 121205 Russia; 5 Department of Clinical Laboratory, Shanghai Children’s Hospital, School of Medicine, Shanghai Jiao Tong University, Shanghai, 200062 China

**Keywords:** H3K4me3, H3K27ac, ChIP-seq, Strand-specific RNA-seq (ssRNA-seq), Prefrontal cortex (PFC), Transcription factor (TF), Histone-modifying enzyme

## Abstract

**Background:**

Changes in gene expression levels during brain development are thought to have played an important role in the evolution of human cognition. With the advent of high-throughput sequencing technologies, changes in brain developmental expression patterns, as well as human-specific brain gene expression, have been characterized. However, interpreting the origin of evolutionarily advanced cognition in human brains requires a deeper understanding of the regulation of gene expression, including the epigenomic context, along the primate genome. Here, we used chromatin immunoprecipitation sequencing (ChIP-seq) to measure the genome-wide profiles of histone H3 lysine 4 trimethylation (H3K4me3) and histone H3 lysine 27 acetylation (H3K27ac), both of which are associated with transcriptional activation in the prefrontal cortex of humans, chimpanzees, and rhesus macaques.

**Results:**

We found a discrete functional association, in which ^H3K4me3^HP gain was significantly associated with myelination assembly and signaling transmission, while ^H3K4me3^HP loss played a vital role in synaptic activity. Moreover, ^H3K27ac^HP gain was enriched in interneuron and oligodendrocyte markers, and ^H3K27ac^HP loss was enriched in CA1 pyramidal neuron markers. Using strand-specific RNA sequencing (ssRNA-seq), we first demonstrated that approximately 7 and 2% of human-specific expressed genes were epigenetically marked by ^H3K4me3^HP and ^H3K27ac^HP, respectively, providing robust support for causal involvement of histones in gene expression. We also revealed the co-activation role of epigenetic modification and transcription factors in human-specific transcriptome evolution. Mechanistically, histone-modifying enzymes at least partially contribute to an epigenetic disturbance among primates, especially for the H3K27ac epigenomic marker. In line with this, peaks enriched in the macaque lineage were found to be driven by upregulated acetyl enzymes.

**Conclusions:**

Our results comprehensively elucidated a causal species-specific gene-histone-enzyme landscape in the prefrontal cortex and highlighted the regulatory interaction that drove transcriptional activation.

**Supplementary Information:**

The online version contains supplementary material available at 10.1186/s12915-023-01612-3.

## Background

Compared with other primates, humans not only have a relatively large brain compared to their weight [1], but also showcase a significant leap in functional complexity such as cognition, thinking, and communication [2, 3]. However, the difference in DNA sequences between humans and their nearest relative species, chimpanzee, is only 1.2%, considering that the two diverged approximately 6–8 million years ago [4–6]. Given this estimated divergence, a crucial question arises: How does human cognitive ability develop during such a short evolutionary time? Changes in gene expression levels have long been thought to play an important role in human evolution [7, 8]. Indeed, when comparing adult humans with other primates, such as chimpanzees and rhesus macaques, excessive human-specific expression changes are observed in genes in the cerebral cortex [8, 9]. Given that this overexpression change has not been found in other sites, such as the blood and liver, gene expression changes in human brains are thought to serve as the basis underpinning human cognitive ability [10]. Besides, previous studies also revealed that genes expressed in the brain accumulated more changes and displayed a 3–5 times accelerated evolutionary pace of developmental pattern divergence on the human than on the chimpanzee lineage [11, 12].

Human cognitive ability and brain development are parallel during the course of development [13]. Humans, especially infants and children, learn and absorb cultural knowledge in the same group as a fundamental basis for their cognitive ability. However, humans and other primates demonstrate considerable differences in cultural knowledge absorption as early as infancy [14]. Accordingly, it is speculated that the developed cognitive ability of humans during an individual’s development is probably a manifestation of specific anatomical and tissue characteristics during brain development. Moreover, these specific anatomical and organizational features are probably reflected in changes at molecular levels specific to human brain development, such as gene expression and splicing, protein and metabolite concentrations, and even epigenetic modification states.

Previous studies have shown that > 15% of genes in the prefrontal cortex (PFC) of human and rhesus macaques showed different developmental expression patterns [15]. By combining microarray and high-throughput sequencing techniques, most of the genes in the PFC and cerebellar cortex of humans, chimpanzees, and rhesus macaques were found to vary significantly with age [12]. Although this change with age is conserved in primate brains, nearly a thousand genes have been identified to exhibit human-specific expression profiles, most of which are present in the early stage of the prefrontal cortex, with a gene count four-fold higher than that of chimpanzees [12, 16]. Additionally, many of these genes are closely linked to the nervous system. The difference is probably a reflection of the extreme delay in human synaptic development [16]. This extreme developmental delay is thought to provide sufficient time for infants to build a more complex neural network than other primates, thus laying the foundation for human cognitive capacity. These studies show the importance of comparing differences in brain developmental expression patterns between humans and other primates to understand human cognitive evolution. Accordingly, elucidating the molecular regulation mechanism of brain-specific developmental expression patterns not only can provide a higher level of understanding for human cognitive evolution but also has a more practical guiding significance for social activities. This is because the healthy development of the human brain is essential for shaping cognitive ability, while its disturbed development leads to severe cognitive impairment and mental retardation, such as autism and schizophrenia (SZ) [17]. Currently, humans suffering from development-related cognitive disorders account for 5–10% of the population worldwide [18]. Such individuals are likely to require medical care for most of their lives, which represents a considerable burden on both their families and society. Studying the molecular regulation mechanism of human brain-specific gene expression helps to reveal the origin of human cognitive ability and provides a valuable theoretical basis for targeted therapy for diseases associated with brain development.

Thus far, some preliminary findings have revealed the molecular regulation mechanism of human cognitive ability formation. In the search for *cis*-regulatory elements, scanning regulatory regions such as promoters and enhancers at the genome-wide level has led to the identification of numerous human-specific mutations in the vicinity of genes involved in nervous system development or brain-specific expression [19, 20]. In the search for *trans*-regulatory elements, although few microRNAs and transcription factors (TFs) have been found, the TFs MEF2A (myocyte-specific enhancer factor 2A) and ERG1–3 (early growth response protein 1–3) represent some examples [12, 16]. Consistent with functional analysis, these TFs regulate the nervous system, including neural survival and synaptic transmission [21–23], further suggesting their important regulatory role in the formation of human cognitive ability.

To date, hundreds of human brain-specific gene expression changes can be explained by a limited number of microRNAs, TFs, mutations in regulatory elements, or epigenetic changes [12, 16, 24–27]. It is worth noting that histone modification can directly regulate the condensed state of chromatin and thus affect gene expression [26, 28–31]. Histone modification has long been thought to be closely related to the healthy development of brain, aging, and cognitive disorders [32–35]. Previous studies have found that histone modification status changes significantly in the brains of primates and patients with cognitive disorders. For example, histone H3K4me3 was modified in the forebrain of newborns [36]. Moreover, the altered state of histone H3K4me2 showed differences in the PFC of rhesus macaques at different ages [37]. Another study identified hundreds of brain-specific modification sites and dozens of brain-specific modification-loss sites by comparing differences in histone H3K4me3 levels in adult humans, chimpanzees, and rhesus macaques [24]. However, human brain cognitive development is a systematic process, and these studies are often limited to a single modified state or a single species, and lack comparison with other molecular levels. Thus, the histone regulatory mechanism of specific gene expression in human brains still remains elusive.

In this study, we measured the genome-wide distribution of H3K4me3 and H3K27ac in the PFC of human, chimpanzee, and rhesus macaque brains, and identified species-specific histone modification sites. By integrating the transcriptome data of humans, chimpanzees, and rhesus macaques derived from the same biological samples, a histone modification regulatory network for the specific expression of genes in the human brain was constructed and its key regulatory factors were identified. Our results significantly contribute to the systematic understanding of molecular regulatory mechanisms of human cognitive ability.

## Results

### H3K4me3 and H3K27ac landscapes of the PFC

**Fig. 1:**
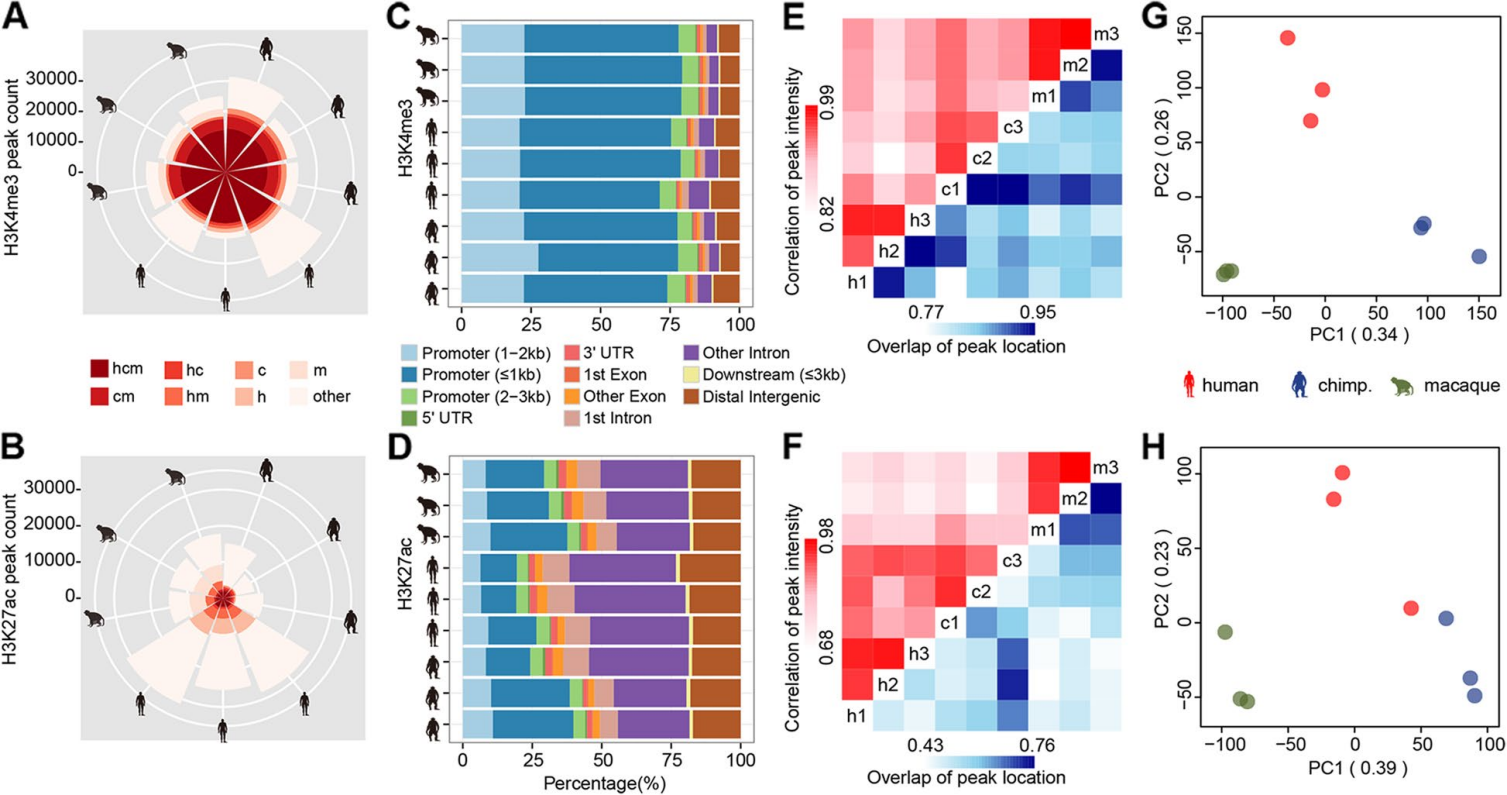
Epigenome profiling in the PFC cortex. **A**, **B** Graphical representation of the global peak distribution. hcm: Peaks shared among all three primates; cm, hc, hm: Peaks shared between pairwise primates; c, h, m: Peaks unique to each primate. The number of peaks is shown on the *y*-axis (*n* = 3) (See replicate data in Additional file 1: Tables S3 and S4). **C**, **D** Overlap of peaks within each individual and given features based on genomic coordinates. Different colors denote different genomic features. The *x*-axis denotes the proportion of peaks intersected with specific genomic features. The *y*-axis denotes each primate (*n* = 3) (See replicate data in Additional file 1: Tables S3 and S4). **E**, **F** Hierarchical clustering of three humans, three chimpanzees, and three macaques based on either correlation of peak intensity (top left) or overlap of peak coordinates (bottom right) for H3K4me3 (**E**) and H3K27ac (**F**) epigenome (*n* = 3) (See replicate data in Additional file 1: Tables S3 and S4). **G**,** H** Principal components based on the peak intensity of H3K4me3 (**G**) and H3K27ac (**H**). Each point represents an individual; colors represent primates (red, human; blue, chimpanzee; green, macaque). The proportion of variance explained by each component is shown on the axis labels (*n* = 3) (See replicate data in Additional file 1: Tables S3 and S4)

In this study, we focused on the dorsolateral PFC, which is involved in cognitive operations that are important for informed choice and creativity among other executive functions and represents a highly associated cortex that is subjected to a disproportionate morphological expansion during primate evolution. Prefrontal H3K4me3 and H3K27ac epigenomes from the gray matter of three adult humans, three adult chimpanzees, and three adult rhesus macaques were measured (Additional file 1: Table S1). The Illumina HiSeq 2000 sequencing platform was used to obtain 20,520,716 to 33,844,133 raw reads for each sample from the H3K4me3 data and 24,548,467 to 29,119,905 raw reads for each sample from the H3K27ac data (Additional file 1: Table S2). MACS (Model-based Analysis of ChIP-Seq) software was used to identify 21,864–37,423 raw peaks for the H3K4me3 epigenome and 8868–31,391 raw peaks for the H3K27ac epigenome (Fig. 1A, B). Consistently, H3K4me3 was mainly enriched around the transcriptional start site (TSS) of active genes and H3K27ac preferentially resided in the promoter and enhancer region. The vast majority (78.3–87.1%) of H3K4me3 peaks were located proximal to the TSSs (within 3 kb) of annotated genes (Fig. 1C), whereas more than 20% of H3K27ac peaks were positioned within 3 kb of known TSSs (Fig. 1D). Peaks with a shared locus were combined to obtain a total of 25,929 H3K4me3 peaks and 14,617 H3K27ac peaks (Additional file 1: Tables S3 and S4). Among them, approximately 60% of the H3K4me3 peaks (15,452 of 25,929) were shared between humans and chimpanzees, with more than 56% of H3K4me3 peaks (14,533 of 25,929) shared across the three species (Fig. 1A). A similar result was observed in the H3K27ac data, in which approximately 18% of H3K27ac peaks (2625 of 14,617) were shared between humans and chimpanzees, with more than 14% of H3K27ac peaks (2049 of 14,617) shared across the three species (Fig. 1B). Moreover, either hierarchical clustering (Fig. 1E, F), by comparing their peak coordinates and peak intensity, or principal component analysis (Fig. 1G, H), based on normalized read counts for all samples, consistently revealed the highest similarities in PFC epigenomes among intra-species and separation trends among inter-species. The Pearson correlation coefficients of the peak locations ranged from 0.846 to 0.948 for H3K4me3 peaks and 0.461–0.759 for H3K27ac peaks within species, while the Pearson correlation coefficients of the peak intensity ranged from 0.944 to 0.989 for H3K4me3 peaks and 0.893–0.977 for H3K27ac peaks within species (Fig. 1E, F).

### Human-specific signatures of the H3K4me3 epigenome

**Fig. 2:**
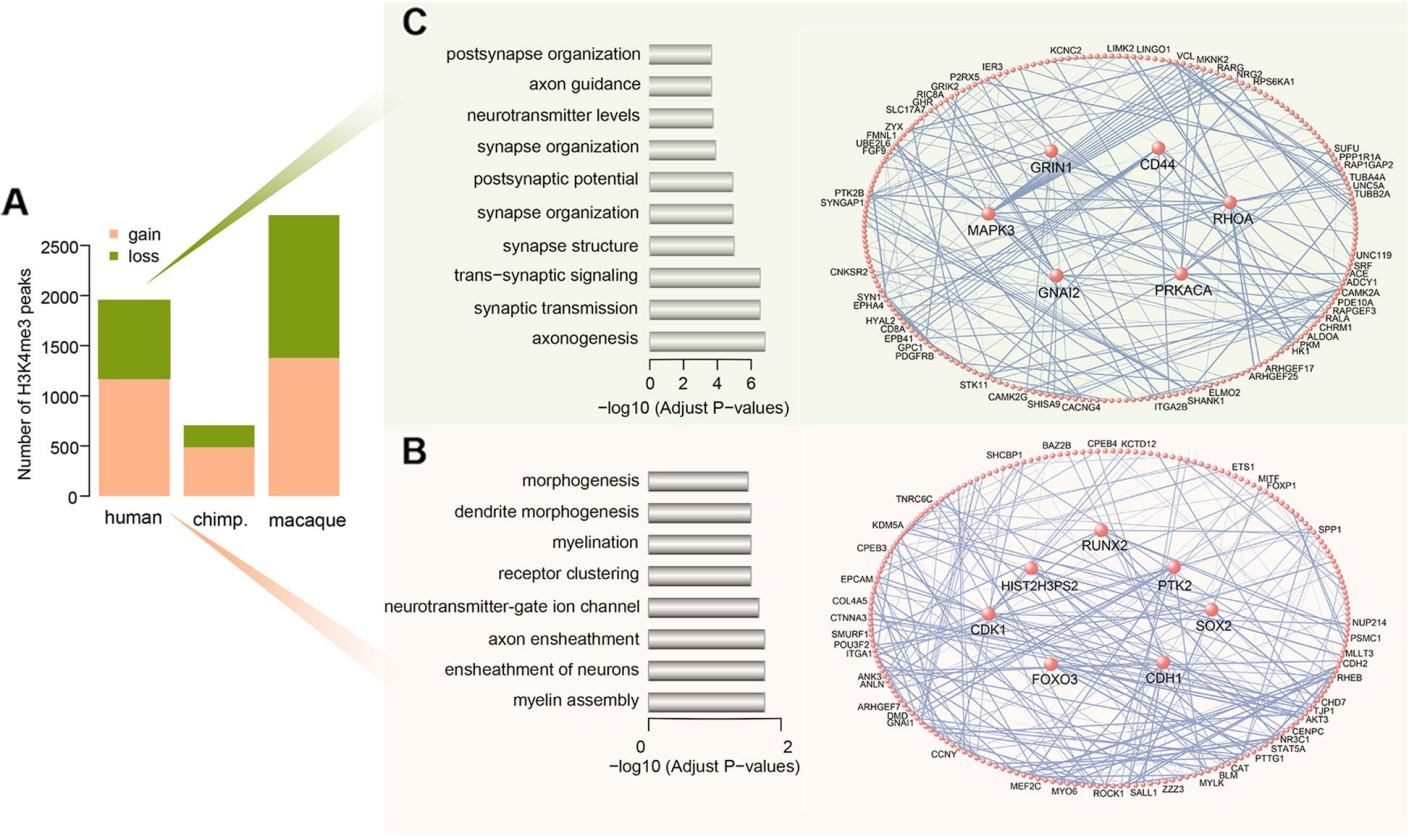
Human-specific signature of the H3K4me3 epigenome. **A** Number of species-specific H3K4me3 peaks. The orange represents H3K4me3 peaks with significant enrichment unique to each primate; green represents H3K4me3 peaks with significant depletion unique to each primate (*n* = 3) (See replicate data in Additional file 1: Tables S5 and S6). **B** Left panel: Gene ontology categories enriched with annotated genes adjacent to ^H3K4me3^HP gain. The *x*-axis denotes log_10_-transformed BH-corrected *P* values. Right panel: Network visualization of genes adjacent to ^H3K4me3^HP gain. **C** Left panel: Gene ontology categories enriched with annotated genes adjacent to ^H3K4me3^HP loss. The *x*-axis denotes log_10_-transformed BH-corrected *P* values. Right panel: Network visualization of genes adjacent to ^H3K4me3^HP loss. For these two networks, the largest sub-networks are shown. Each circle represents an individual gene. Genes with the highest connectivity (i.e., hubs) are shown as larger sizes

To identify loci with human-specific H3K4me3 and H3K27ac signatures in PFC, we screened 25,929 H3K4me3 peaks and 14,617 H3K27ac peaks detected in primates. Using the “edgeR” package, we identified 2396 peaks significantly enriched in human samples as compared with the two non-human primates after correcting for false discovery rate (FDR). We further grouped the human-specific H3K4me3 peaks into upregulated and downregulated categories, with histone modification levels that were at least 1.2-fold increased or decreased in humans compared to those in chimpanzees and macaques. In total, we obtained 1175 upregulated H3K4me3 peaks and 775 downregulated H3K4me3 peaks in humans. In parallel, 487 upregulated H3K4me3 peaks and 219 downregulated H3K4me3 peaks in the chimpanzee were obtained. The H3K4me3 peaks were specifically upregulated or downregulated in humans approximately 2.4 and 3.5 times, respectively, more than those in the chimpanzee. Hereafter, we use the term “^H3K4me3^HP” to denote “human-specific H3K4me3 peaks” for further analysis (Fig. 2A; Additional file 1: Tables S5 and S6). Strikingly, the genes adjacent to ^H3K4me3^HP gain regions showed highly significant enrichment for genes involved in myelination assembly, neuronal ensheathment, receptor clustering, and high overlap with oligodendrocyte markers (48/365 genes, BH-corrected *P* = 1.905 × 10^−15^), all of which were related to myelin membrane formation and signaling transmission (Fig. 2B; Additional file 1: Table S7). The hubs with ^H3K4me3^HP gain regions, that is, the genes proximal to ^H3K4me3^HP gain regions with the highest connectivity, were RUNX2, SOX2, FOXO3, CDK1, CDH1, PTK2, and HIST2H3PS2 (Fig. 2B). RUNX2, SOX2, and FOXO3 are well-recognized TFs required for the self-renewal of neural stem and progenitor cells, thereby promoting the cell cycle in proliferating progenitors [38–42]. CDK1, CDH1, PTK2, and HIST2H3PS2 are involved in cell cycle progression, cellular adhesion, and proliferation [43–45]. Moreover, the genes adjacent to the ^H3K4me3^HP loss regions were enriched for CA1 pyramidal neuron markers (26/329 genes, BH-corrected *P* = 6.927 × 10^−4^) and markers for S1 pyramidal neurons (17/226 genes, BH-corrected *P* = 9.855 × 10^−3^), as well as for genes associated with synaptic transmission and axonogenesis, all of which are related to synaptic activity (Fig. 2C; Additional file 1: Table S8). Consistent with this functional annotation, two of the hubs were MAPK3 and GRIN1, which play a vital role in the plasticity of synapses, thereby contributing to memory and learning (Fig. 2C) [46, 47]. Another four of the hubs were CD44, RHOA, GNAI2, and PRKACA, all with active roles in transmembrane activities [48–51].

### Human-specific signatures of the H3K27ac epigenome

**Fig. 3:**
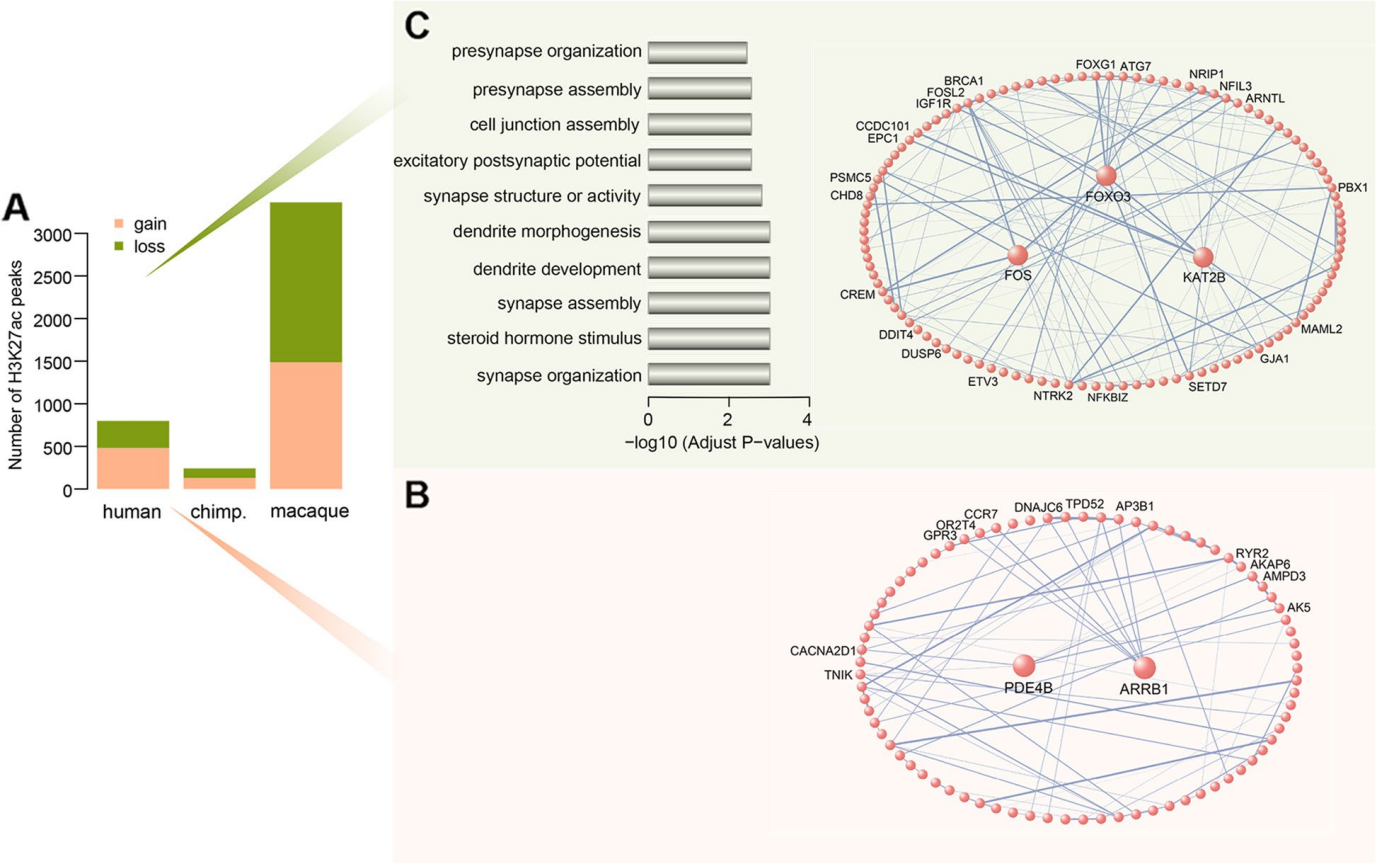
Human-specific signature of the H3K27ac epigenome.** A** Numbers of species-specific H3K27ac peaks. The orange represents H3K27ac peaks with significant enrichment unique to each primate, green represents H3K27ac peaks with significant depletion unique to each primate (*n* = 3) (See replicate data in Additional file 1: Tables S9 and S10). **B** Network visualization of genes adjacent to ^H3K27ac^HP gain. **C** Left panel: Relevant gene ontology categories enriched with annotated genes adjacent to ^H3K4me3^HP loss. The *x*-axis denotes log_10_-transformed BH-corrected *P* values. Right panel: Network visualization of genes adjacent to ^H3K27ac^HP loss. The largest sub-networks are shown for both networks. Each circle represents an individual gene. Genes with the highest connectivity (i.e., hubs) are shown as larger sizes

We identified 799 H3K27ac peaks (gain: 483; loss: 316) and 242 H3K27ac peaks (gain: 131; loss: 111) that showed significant changes in human and chimpanzee PFCs, respectively. Slightly different from the observation of H3K4me3 modification, the H3K27ac peaks that were specifically upregulated or downregulated in humans were approximately 3.7 and 2.8 times, respectively, more than those in the chimpanzee. Hereafter, we use the term “^H3K27ac^HP” to denote “human-specific H3K27ac peaks” for further analysis (Fig. 3A; Additional file 1: Tables S9 and S10). Among the genes close to ^H3K27ac^HP gain regions, two hubs (PDE4B and ARRB1) were known to mediate cellular response to extracellular stimuli, inferring functional relevance based on a network approach (Fig. 3B) [52, 53]. Additionally, these genes remarkably showed overrepresentation in interneuron markers (12/302 genes, BH-corrected *P* = 6.317 × 10^−4^) and markers of oligodendrocytes (11/365 genes, BH-corrected *P* = 6.422 × 10^−3^) (Additional file 1: Table S11). For the genes adjacent to ^H3K27ac^HP loss regions, all of which showed highly significant enrichment for many tightly related gene ontology categories, including synapse organization and activity, and were enriched for CA1 pyramidal neuron markers (13/329 genes, BH-corrected *P* = 3.133 × 10^−4^), and thus played a critical role in neuronal signal transduction (Fig. 3C; Additional file 1: Table S11). FOS, one of the hubs, is currently regarded as a marker of neuronal activity and has been associated with neural and behavioral responses to extracellular stimuli [54]. KAT2B is a lysine histone acetyltransferase highly expressed in the brain, which has highlighted its vital importance for brain function and proper development [55].

### Regulation of the human-specific gene pattern by ^H3K4me3^HP and ^H3K27ac^HP

**Fig. 4:**
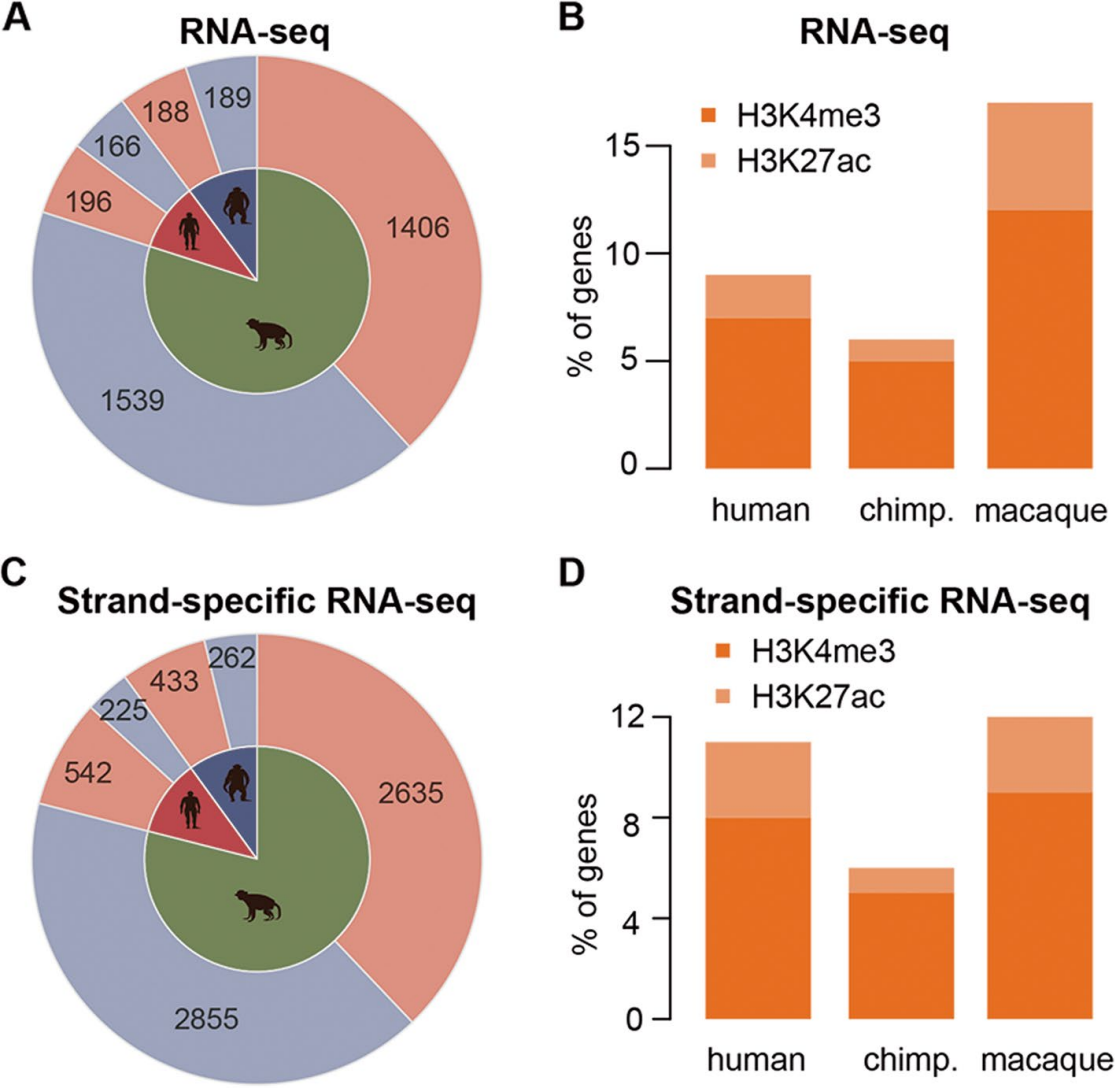
Regulation of species-specific gene expression by species-specific H3K4me3 and H3K27ac peaks. **A**, **C** Summary of the species-specific expressed genes detected in the RNA-seq dataset (**A**) and ssRNA-seq dataset (**C**). The pink and purple denote upregulated and downregulated genes specific to primates, respectively. The silhouette denotes primate identity throughout the figure (red, human; blue, chimpanzee; green, macaque). Digits in areas denote the number of genes. **B**, **D** Proportion of species-specific expressed genes regulated by species-specific H3K4me3 peaks (orange) and H3K27ac peaks (light orange) in the RNA-seq (**B**) and ssRNA-seq (**D**) datasets. The *y*-axis displays percentile values. **C**, **D** (*n* = 3) (See replicate data in Additional file 1: Table S13)

To determine the consequences of epigenetic divergence on gene expression divergence across the three primates, we first tested the fraction of human-specific expressed genes subjected to ^H3K4me3^HP and ^H3K27ac^HP in PFC, respectively. We used transcript levels measured by RNA-seq in the PFC of ten humans, eight chimpanzees, and seven rhesus macaques published previously [56–59]. We re-analyzed the 12,464 genes expressed among all three adult primates, of which, 362 genes were differentially expressed between humans and the other two primates (Fig. 4A). It has been reported that *cis*-elements accumulate in the regulatory region over time and tend to affect each gene independently. Unsurprisingly, approximately 7% of human-specific expressed genes were correlated with ^H3K4me3^HP, whereas approximately 2% were correlated with ^H3K27ac^HP (Fig. 4B). This observation may be attributed to the fact that approximately a 2.5-fold excess of H3K4me3 peaks showed human-specific histone modification compared to H3K27ac peaks, thereby shaping transcriptome divergence by epigenetic modification in H3K4me3.

To test whether these findings were replicable and to further validate the results in an independent dataset, we obtained 1062 ^H3K4me3^HP (gain: 885; loss: 177) with at least 1.5-fold differential tag density in nine adult humans and compared them to three macaques and four chimpanzees from the published epigenetic data, which were detected using an independent Poisson statistic [24]. As a result, approximately 7% of human-specific expressed genes were enriched with ^H3K4me3^HP, which agrees with our measurement. Moreover, for H3K27ac, we re-analyzed an additionally published epigenome from the PFC region of three humans, two chimpanzees, and three macaques [60]. Of the total 60,702 H3K27ac peaks, 933 ^H3K27ac^HP (gain: 428; loss: 505) were detected under the same filtering criteria (BH-corrected *P* < 0.05, fold change > 1.2) with the same statistical procedure—the quasi-likelihood methods implemented in the edgeR package. Consequently, approximately 3% of human-specific expressed genes were enriched with ^H3K27ac^HP. Considering peaks up to four-fold greater than ours, when shrunken to a more stringent criterion, i.e., fold change > 2, the number of ^H3K27ac^HP decreased to 740 (gain: 284; loss: 456). Consequently, approximately 2% of human-specific expressed genes were found to co-localize with ^H3K27ac^HP. Therefore, independent analyses produced robust and reproducible results.

It has been previously reported that strand-specific RNA-seq (ssRNA-seq) provided more reliable resolution of transcriptome profiling and more accurate measurement of gene expression [61]. Here, we collected the same cohort of three humans, three chimpanzees, and three macaques from PFC neurons measured using the ssRNA-seq technique. We obtained 40,680,796 to 59,816,568 raw reads for each sample from the ssRNA-seq data (Additional file 1: Table S12) and screened 28,850 genes sharing comparable expression patterns across three primates (Additional file 1: Table S13). A total of 767 genes (up: 542; down: 225) were determined to be differentially expressed between humans and the other two primates (Fig. 4C). Remarkably, the species-specific genes overlapped significantly with their counterparts reported in the RNA-seq dataset (Fisher’s exact test, *P* < 0.001; Additional file 2: Fig. S1A). Furthermore, the expression changes specific to each species observed both in the RNA-seq and ssRNA-seq datasets correlated very well (Pearson correlation coefficient, *r* = 0.77 ± 0.13), despite the only partial overlap of genes between studies (Additional file 2: Fig. S1B). Albeit marginally, approximately 8 and 3% of human-specific expressed genes were enriched with ^H3K4me3^HP and ^H3K27ac^HP, respectively (Fig. 4D).

To further estimate whether the species-specific histone modification affects the genes that showed changes in expression during development, we tested the overlap between genes related to a species-specific histone modification and three types of genes identified in a previous study [12]: genes with constant expression across the lifespan (type I), genes showing variable expressions across lifespan but no developmental pattern differences among species (type II), and genes with developmental remodeling within species (type III). All three types were found to be profoundly enriched with ^H3K4me3^HP, while ^H3K27ac^HP appeared to show a significant overrepresentation of type II and type III genes (Additional file 2: Fig. S2). Consequently, the species-specific histone modification not only affected the genes with expression changes in primates but also with the developmental remodeling of their expression patterns in primates. These findings suggest that human-specific histone modification contributes significantly to gene expression evolution in primate PFC.

### Histone-TF target regulatory network

**Fig. 5:**
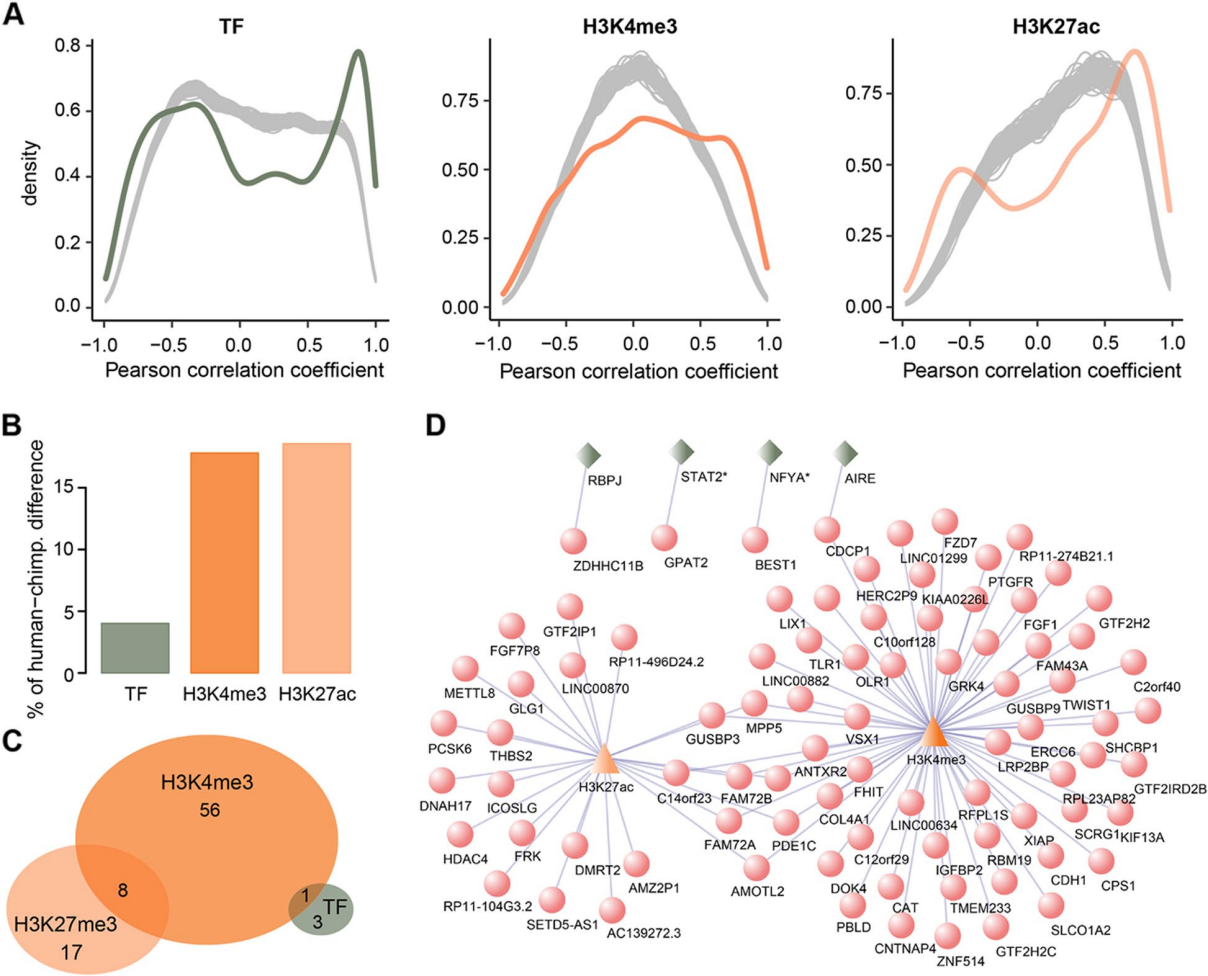
Histone-TF target regulatory network.** A** Colored curves showing the correlation between species-specific expressed genes detected in the ssRNA-seq dataset and their corresponding regulators. The gray areas show the expected by chance correlation calculated by randomly sampling the same number of non-species-specific genes and their coupled regulators based on 100 permutations. The *x*-axis denotes the Pearson correlation coefficients and the *y*-axis denotes the density estimation. **B** The percentage of gene expression variance between humans and chimpanzees explained by TF expression differences, or H3K4me3 and H3K27ac coverage differences. **C** The overlap of the human-specific genes that significantly correlated with TFs, or those close to ^H3K4me3^HP and ^H3K27ac^HP. **A–C** Green, TF; orange, H3K4me3; light orange, H3K27ac. **D** Network visualization of human-specific genes regulated by TF, ^H3K4me3^HP, and ^H3K27ac^HP corresponding to **C**. Genes marked with asterisks denote TFs highly correlated with human-specific genes. Red circle, human-specific gene; triangle, H3K4me3 (orange) and H3K27ac (light orange); green quadrangle, TF

We hypothesized that the human-specific gene expression in the PFC is possibly driven either by an orchestrated interplay of *cis*-elements and *trans*-factors or independently. To this end, we focused on 1950 ^H3K4me3^HP and 799 ^H3K27ac^HP from our dataset, as well as 211 TFs from a previously published study [15]. As expected, TF, H3K4me3, and H3K27ac significantly correlated with a combination of three species-specific genes detected in our ssRNA-seq dataset (Fig. 5A). Next, we investigated the relationship between gene expression differences and corresponding TF expression differences, as well as the relationship between gene expression differences and coupled epigenetic differences between humans and chimpanzees by fitting a linear regression model. To avoid bias, only those TFs that were strongly correlated with the expressed genes, i.e., absolute Pearson correlation coefficient > 0.6 and *P* < 0.05, were used in the model. In total, we identified 69 TFs with at least one target gene that was human- or chimpanzee-specific (Additional file 1: Table S14). Among them, five most relevant TFs showed both positive and negative correlations with their regulatory target genes, including SREBF1 downregulated with Alzheimer’s disease (AD) in oligodendrocytes [62], POU2F1 as a potential regulator of excitatory neuron development in a mouse model [63], PAX8 increased survival and immortalization in gliomas cells [64], POU5F1 (also known as OCT4), its ectopic expression, combined with SOX2 and KLF4, promoted axon regeneration after injury [65], and MEIS1 involved in the development of the central nervous system and considered to be the strongest genetic risk factor for restless legs syndrome [66]. Consequently, we found a significant positive correlation between the three regulators and human-chimpanzee gene expression differences (permutation test, *P* < 0.001; Additional file 2: Fig. S3A), which was presumably due to the transcriptional activation function of TFs and histones. TFs explained approximately 4% of the gene expression differences between humans and chimpanzees; the H3K4me3 and H3K27ac peaks located close to human- and chimpanzee-specific genes explained as high as 17.8 and 18.6% of the gene expression differences, respectively, which were profoundly larger than those to be expected by chance (permutation test, *P* < 0.001; Fig. 5B and Additional file 2: Fig. S3B). This result raised the possibility that histone H3 lysine 4 trimethyl and lysine 27 acetyl modification contributed to a wider extent of gene expression variation between humans and chimpanzees.

To assess the interplay of various regulatory mechanisms specific to the human lineage, we coupled TFs to human-specific expressed genes and found that the expression of six TFs was significantly positively or negatively correlated with the expression of their corresponding target genes (one-sided Wilcoxon rank-sum test, BH-corrected *P* < 0.05; Pearson correlation coefficient, |*r*|> 0.6) than that expected by chance (permutation test, *P* = 0.015; Additional file 2: Fig. S3C). Considering that a single TF can regulate multiple expressed genes simultaneously, one gene probably possesses multiple transcription factor binding sites (TFBS) and can recruit several TFs to control its expression. We then assessed the putative human-specific genes that were strongly correlated with TFs by comparing the absolute Pearson correlation coefficients between the expression profile of the gene and corresponding TFs to those calculated between the same gene and the same number of TFs that were randomly selected from all expressed genes using a one-sided Wilcoxon rank-sum test based on 1000 permutations. Under the criteria of BH-corrected *P* < 0.05 and absolute Pearson correlation coefficient > 0.6, four human-specific genes were estimated to strongly correlate with TFs (permutation test, *P* < 0.001; Additional file 2: Fig. S3C). Collectively, of 64 (8%) human-specific genes enriched with ^H3K4me3^HP, eight expressed genes were also enriched with ^H3K27ac^HP, and only one gene was co-regulated by ^H3K4me3^HP and TFs (Figs. 4D and 5C, D). Notably, our results indicated that ^H3K27ac^HP and TFs behaved in a mutually exclusive manner.

There is extensive evidence suggesting that one of the human-specific genes CDCP1, collaboratively regulated by ^H3K4me3^HP and TF, may act as a novel regulator and promote the proliferation and migration of glioma, which is a primary, malignant, and aggressive brain tumor in adults [67]. Interestingly, as a pro-inflammatory cytokine, the reduced expression of CDCP1 is demonstrated to be protective in autoimmune encephalomyelitis [68]. The expression of transcription factor STAT2 was determined to be significantly correlated with the human-specific genes detected in our ssRNA-seq dataset, while the overexpression of its inhibitor PIAS2 has been reported to cause motor and cognitive impairments, predisposing one to sporadic Parkinson’s disease [69]. Moreover, our results showed that nuclear transcription factor Y subunit alpha (NFYA), a key regulator of cell cycle progression, correlated strongly with human-specific genes. Interestingly, Yamanaka et al*.* proposed that the suppression of neuronal NFYA using gene deletion or knockdown strategies resulted in progressive neurodegeneration [70].

Taken together, these results support that the overwhelming majority of human-specific genes are predominantly regulated by human-specific *cis*-elements alone rather than the crosstalk of *cis*-elements and *trans*-factors.

### Assessment of enzymatic intervention in histone modification changes

**Fig. 6:**
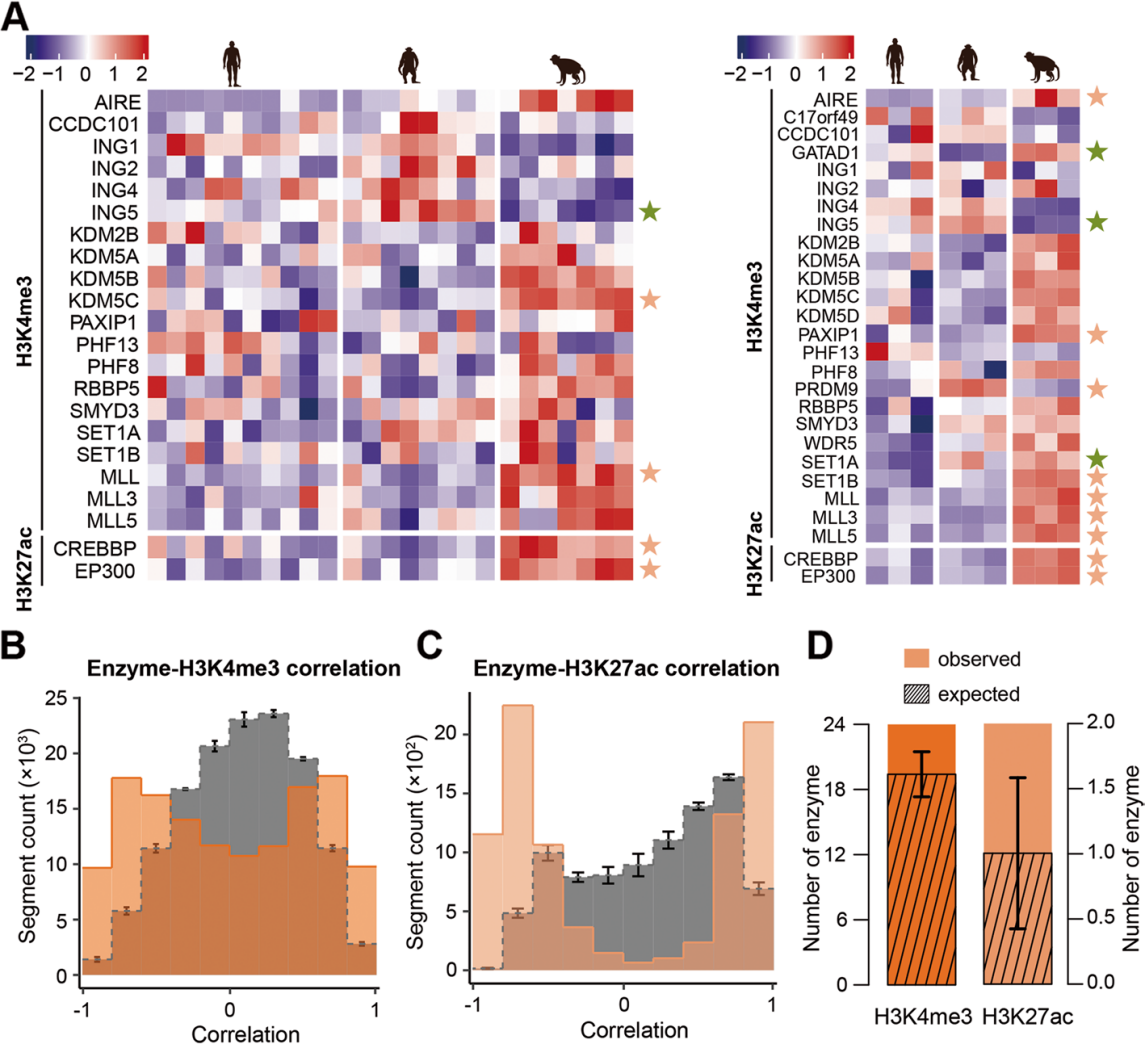
Expression profile of histone-modifying enzymes.** A** Heatmap of 22 histone-modifying enzymes based on gene expression levels measured in the RNA-seq dataset (left panel) and 27 histone-modifying enzymes based on the gene expression levels measured in the ssRNA-seq dataset (right panel). Corresponding human, chimpanzee, and macaque as indicated (top). Color scaled bars represent the normalized read counts. Stars on the right side denote species-specific expressed genes. Human-specific genes: SET1A; chimpanzee-specific genes: GATAD1, PRDM9; macaque-specific genes: AIRE, ING5, PAXIP1, KDM5C, SET1B, MLL, MLL3, MLL5, CREBBP, EP300. (orange: upregulated; green, downregulated). **B** Pearson correlation of 25 H3K4me3-modifying enzymes derived from ssRNA-seq dataset and 5459 species-specific H3K4me3 peaks. **C** Pearson correlation of two H3K27ac-modifying enzymes derived from ssRNA-seq dataset and 4404 species-specific H3K27ac peaks. **B**, **C** The gray bars represent the mean correlation coefficients expected by chance, calculated by randomly subsampling the same number of non-species-specific peaks and repeating the permutation 100 times. The test was conducted using the expression profiles for all three species. The error bars represent the mean ± 3SD. **D** Number of enzymes significantly correlated with H3K4me3 and H3K27ac peaks (colorful bars). The Pearson correlation of each enzyme derived from ssRNA-seq dataset and species-specific histone peaks was compared to the correlations between the same enzyme and the same number of non-species-specific peaks using one-sided Wilcoxon test (*P* < 0.01). The average number based on 1000 permutations is defined as the number of significantly correlated enzymes. The streaked bars represent the average number of correlated enzymes expected by chance, calculated from 1000 random samplings of nonenzymatic genes and the same number of non-species-specific histone peaks. The test was conducted using all three species’ expression profiles. The error bars represent the mean ± SD

As histone is susceptible to control by a group of enzymes, we next explored whether histone-modifying divergence occurring in PFC across the three species was attributed to enzymatic activities. For this purpose, we collected 28 H3K4me3-modifying enzymes and two H3K27ac-modifying enzymes from HISTome2 together with the EpiFactors database (Additional file 1: Table S15). As a result, 22 and 27 enzymes were detected in the RNA-seq and the ssRNA-seq dataset, respectively (Fig. 6A). Specifically, CREBBP and EP300, which mediate the acetylation of histone H3 at “Lys-27,” were highly expressed in the macaque lineage compared to the other two primates, and further showed a significant overrepresentation in macaque-specific upregulated genes measured in either the RNA-seq dataset (Fisher’s exact test, *P* = 0.0127) or the ssRNA-seq dataset (Fisher’s exact test, *P* = 0.0083), which indicated that the enrichment of H3K27ac peaks unique to macaque presumably could be driven by upregulated H3K27ac-modifying enzymes. Notably, we observed that the expression profiles of enzymes showed a profoundly higher correlation with 5459 species-specific H3K4me3 peaks and 4404 H3K27ac peaks than that expected by chance (Figs. 6B, C; permutation test, *P* < 0.001). We also observed a significant excess of correlated H3K4me3- and H3K27ac-modifying enzymes than expected by chance (Fig. 6D; permutation test, *P* < 0.001). Cumulatively, these findings support that enzymatic expression, to some extent, facilitates the explanation of histone-modifying divergence among species.

## Discussion

The human brain and cognitive abilities develop in parallel throughout ontogenesis, resulting in a phenotype strikingly distinct from that of other primates. Previous studies have suggested that remodeling of the gene expression trajectory during brain development plays a crucial role in human cognitive evolution [10, 71–73]. However, the regulatory mechanisms that are responsible for gene expression changes during human brain development are unclear. It must be noted that histone modifications, which shape gene expression patterns, affect neuronal functions in both healthy and diseased brains. Thus, understanding the epigenetic regulatory mechanisms of gene expression changes during human brain development is essential to understanding the origin of cognitive evolution and development-related mental disorders. In this study, we mapped genome-wide H3K4me3 and H3K27ac in the PFC of human and two non-human primates, including closely related living primate relatives -chimpanzees and monkey-rhesus macaques. In total, 25,929 H3K4me3 peaks and 14,617 H3K27ac peaks were consistently detected in at least one species. By grouping histone modification peaks into species-specific categories, approximately 2.8 and 3.3 times more H3K4me3 and H3K27ac were detected in humans, respectively, compared to the chimpanzee. Furthermore, either H3K4me3 or H3K27ac peaks with specific patterns identified in humans are widely associated with genes related to cognitive function.

An analysis of functional interrogation revealed that ^H3K4me3^HP gain regions were enriched for genes involving myelination assembly, neuronal ensheathment, and receptor clustering. They also showed a strong overlap with genes that were specifically expressed in oligodendrocytes. Oligodendrocytes, constituting 50–75% of the glial cells in the neocortex, provide metabolic and trophic support to axons and have been implicated in the cellular phase of AD [74, 75]. ^H3K4me3^HP loss regions showed significant enrichment for CA1 and S1 pyramidal neurons, as well as gene categories of synaptic transmission and axonogenesis. Of note, the hippocampus, the origin of CA1 pyramidal neurons, is central to learning and memory functions and points to the acceleration of transcriptomic evolution in humans compared to other primates [76, 77]. Furthermore, a previous study demonstrated the simultaneous tight interconnection of CA1 and S1 pyramidal neurons by biclustering genes and cells, suggesting the orchestration of functionality [78]. Interestingly, the ^H3K27ac^HP gain regions were revealed to be enriched in interneuron and oligodendrocyte markers. This result is notable because oligodendrocytes and axons have reciprocal communication, in which oligodendrocytes receive instructive signals from axons that direct their myelination, and subsequently shape axonal structure and conduction. Therefore, this finding highlights that oligodendrocytes provide indispensable support to neurons [79–81]. Interruption of GABAergic signaling to oligodendrocyte precursor cells has been shown to contribute to reduced myelination and hypoactivity of interneurons, as well as significant changes in cortical network activities and impaired social cognitive behavior [82]. Moreover, a series of intensive early studies have reported the disturbed crosstalk of oligodendrocyte-interneuron in AD and SZ, suggesting a novel therapeutic target [83–85]. Parvalbumin interneuron hypomyelination is associated with cognitive inflexibility in these disorders, which is caused by impaired maturation of myelin-producing oligodendrocytes. This notion is accompanied by reduced myelin- and oligodendrocyte-related protein levels in the AD and SZ brains, such as myelin basic protein (MBP), myelin proteolipid protein (PLP), cyclic nucleotide phosphohydrolase (CNP), myelin-associated glycoprotein (MAG), and myelin oligodendrocyte glycoprotein (MOG), indicating a loss of myelin. A pilot study showed a decreased level of contactin-associated protein, which mediates communications between oligodendrocytes and synapses, thus indicating defective oligodendrocyte-neuronal interactions in SZ [86]. The ^H3K27ac^HP loss regions were also found to be closely associated with synapse organization and activity, as well as hippocampal CA1 pyramidal neuron markers, which are central to both the acquisition and maintenance of memory. In the PFC of 48 participants with varying degrees of AD pathology, Mathys et al. identified 1115 differentially expressed genes (DEGs) in cells isolated from AD-pathology versus no-pathology individuals across six major brain cell types [87]. Therefore, this encouraged us to investigate what was the transcriptional regulatory mechanism of H3K4me3 and H3K27ac involved in AD. We observed that ^H3K4me3^HP loss regions were exclusively enriched in excitatory neurons (hypergeometric test, BH-corrected *P* = 3.658 × 10^−8^), which were overwhelmed by downregulated DEGs, reaching up to 75% (Additional file 2: Fig. S4A). Both ^H3K4me3^HP gain and ^H3K27ac^HP loss regions showed strong overrepresentations with oligodendrocyte markers (hypergeometric test, BH-corrected *P* = 5.64 × 10^−7^ for ^H3K4me3^HP gain and BH-corrected *P* = 2.698 × 10^−3^ for ^H3K27ac^HP loss), which comprised 102 upregulated and 71 downregulated DEGs (Additional file 2: Figs. S4A and S4B). These results indicated that histone modification changes in human PFC might be involved in cognitive evolution in the human lineage and cognitive impairment in neurodegenerative disorders.

Based on both routine and ssRNA-seq techniques, we estimated approximately 7 and 2% of human-specific expressed genes compared to non-human primates were co-occurrent with the signals of ^H3K4me3^HP and ^H3K27ac^HP, respectively. As gene expression changes, especially remodeling of the gene expression trajectory during brain development, have been suggested to play an important role in human cognitive evolution, we speculated that histone modification changes in human PFC could contribute to gene expression alterations and evolution of advanced cognition in humans.

It must be noted that our work has several limitations. First, given that the differences between the human and non-human H3K4me3 and H3K27ac landscape are the major axis of epigenomic variation, it would be indispensable for future studies to pursue more samples; such a high-resolution approach is expected to reveal larger numbers of epigenomic loci unique to each primate, and avoid statistical underpower owing to a small sample size. Second, we used adult brains for cross-species comparisons, but human-specific signatures in the neuronal cortex are reported to be even more pronounced during pre- and perinatal development. Accordingly, it is reasonable to assume that younger brains may display changes at additional loci, or more pronounced alterations in the regulatory region of some genes identified in this study. To overcome these limitations, we firstly re-analyzed the histone modification data from published studies [24, 60] and confirmed the proportions of human-specific expressed genes, which might be caused by the modification changes in H3K4me3 and H3K27ac, were generally robust. Although only adult primates were used in this study, using the genes identified to change their expression trajectory during development in human PFC [12], we tested the potential influence of histone modification on developmental remodeling genes. Constitutive expression divergence represented by type I and II genes showing constant expression across lifespan or no developmental pattern changes among species reflected different results; both two types were profoundly enriched with ^H3K4me3^HP, while ^H3K27ac^HP tended to show a significant overrepresentation of type II genes, they have no association with type I genes (Additional file 2: Fig. S2). By contrast, we found genes that underwent developmental remodeling (type III) showed significant associations with ^H3K4me3^HP and ^H3K27ac^HP, accounting for approximately 12 and 5% of type III genes, respectively. This result suggested that the histone modification changes in human PFC not only affect the genes with expression changes in primates but also with the developmental remodeling of their expression patterns in primates. Given the role played by developmental remodeling genes in neurons and neuronal functions [12], it is conceivable that the observed human-specific histone modification changes in the PFC could underlie advanced cognitive abilities in the human brain.

Our findings consolidate the importance of evolutionary changes in *cis*-regulatory mechanisms that drive evolutionary changes in gene expression. The fact that many genes show human-specific expression signatures regulated by histone modification raises questions about the synchronized role of *cis*-elements and *trans*-factors during primate genome evolution. To clarify this issue, additional 211 TFs and their predicted target genes were added. Our results revealed that both TFs and epigenetics correlated well with species-specific gene expression and to some extent could be responsible for the gene expression difference between humans and chimpanzees, with an explained variance ranging from 4 to 18.6%. These observations suggest that both TF and epigenetic modification associated with active transcription contribute to transcriptome evolution among primates. We also identified six TFs that showed a significantly high correlation with some of the human-specific expressed genes detected in the ssRNA-seq dataset, including NFYA, SREBF1, HNF4A, HMGA1, MEIS1, and STAT2. To date, numerous studies have analyzed these TFs in the brain. Indeed, SREBF1 was declared to be an antipsychotic-activated TF controlling cholesterol biosynthesis, which is involved in the etiology of SZ [88]. Morabito and colleagues found that variability in the SREBF1 motif was decreased in late-stage AD. They also revealed that SREBF1 expression was downregulated in AD oligodendrocytes, highlighting a new avenue for AD therapeutics [62]. A network-based meta-analysis identified HNF4A, a TF associated with gluconeogenesis and diabetes, as a central longitudinally dynamic biomarker for Parkinson’s disease [89]. Furthermore, a series of studies have proposed that the impaired expression of HMGA1 in glioma cells may be linked to gliogenesis and confer a survival benefit to mesenchymal glioblastoma stem-like cell (GSC) tumors [90–92]. Moreover, deficiency in the MEIS1 gene has been revealed to explain some of the iron or dopamine changes in relation to restless legs syndrome [93, 94]. Remarkably, our results suggest that human-specific genes are prone to be independently regulated by TFs and epigenetic modification.

Regarding the drivers of this epigenetic evolution, we found that the expression profile of enzymes correlated with species-specific H3K4me3 and H3K27ac epigenomes was significantly better than that expected by chance, suggesting a potential mechanism contributing to genome evolution. Two well-known enzymes associated with H3K27ac, CREBBP and EP300, were found to be highly expressed in the macaque lineage compared to the other two primates and showed remarkable correlation with macaque-specific upregulated genes. This result raised the possibility that the higher epigenomic levels of H3K27ac specific to the macaque may be driven by upregulated H3K27ac-modifying enzymes. Furthermore, several enzymes and enzyme families associated with H3K4me3 showed profoundly higher levels in macaques than in the other two primates; these include, among others, the lysine methyltransferases KMT (a.k.a MLL) family and the lysine demethylases KDM family. The H3K4-specific methyltransferase, MLL1, is essential for cortical and hippocampal development and may play an important role in the etiology of SZ [95–97]. Therefore, it is becoming evident that the epigenome, to some extent, could have evolved as a consequence of enzymatic divergence.

## Conclusions

Our findings, based on a more comprehensive and detailed analysis of specific histone modification profiles, underscore the importance of epigenomic fine mapping for the human brain in determining genome regulation and provide important insights into the possible nature of molecular mechanisms underlying human cognitive evolution.

## Methods

### Collection of brain samples

We used the same cohort of PFC samples from the postmortem brains of three adult humans, three adult chimpanzees, and three rhesus macaques for ChIP-seq and strand-specific RNA-seq (Additional file 1: Table S1). Human samples were obtained from the NICHD Brain and Tissue Bank for Developmental Disorders at the University of Maryland (USA). All subjects were healthy, as defined by forensic pathologists at the tissue bank. Chimpanzee samples were obtained from the Anthropological Institute & Museum of the University of Zürich-Irchel (Switzerland), and the Biomedical Primate Research Centre (Netherlands). Rhesus macaque samples were obtained from the Suzhou Experimental Animal Center (China). All non-human primates used in this study suffered sudden deaths for reasons other than their participation in this study and had no relationship to the collected tissues. The PFC samples were dissected from the anterior part of the superior frontal gyrus, corresponding to the Brodmann area 10. All tissues were snap-frozen after dissection and stored at − 80 °C.

### Chromatin immunoprecipitation sequencing (ChIP-seq)

ChIP was carried out as described previously with some modifications [98]. Briefly, 0.15 g of PFC tissue was ground into powder on dry ice and crosslinked in 1% formaldehyde for 10 min at room temperature. Then, the reaction was quenched by adding glycine, and the tissue was further homogenized in a glass douncer. Cell pellets were lysed successively in Farnham lysis buffer (5 mM PIPES pH 8.0, 85 mM KCl, 0.5% NP-40) and lysis buffer (1% SDS, 10 mM EDTA, 50 mM Tris–HCl, pH 8.0) on ice. Nuclei were sonicated for 5 s at 15% power of Misonix Q700 for 90 times with a 10-s refractory period. The supernatant was diluted in dilution buffer (1.1% Triton X-100, 0.25% sodium deoxycholate, 1.2 mM EDTA, 167 mM NaCl, 16.7 mM Tris–HCl, pH 8.0) and transferred to the Protein A/G magnetic beads which were pre-incubated with H3K4me3 (Millipore, 07–473) or H3K27ac (Active Motif, 39,133) antibody in PBS with 0.5% BSA at 4 °C for at least 4 h, and rotated overnight at 4 °C. The beads were then washed sequentially with low-salt wash buffer (0.1% SDS, 1% Triton X-100, 0.25% sodium deoxycholate, 1 mM EDTA, 150 mM NaCl, 50 mM Tris–HCl, pH 8.0), high-salt wash buffer (0.1% SDS, 1% Triton X-100, 0.25% sodium deoxycholate, 1 mM EDTA, 500 mM NaCl, 50 mM Tris–HCl, pH 8.0), LiCl wash buffer (500 mM LiCl, 1% NP-40, 1% sodium deoxycholate, 100 mM Tris–HCl, pH 8.0), and TE buffer and eluted with elution buffer (1% SDS, 0.1 M NaHCO_3_) at 65 °C. After adding NaCl to the final 200 mM, the chromatin in the supernatant was reverse crosslinked overnight at 65 °C and then treated with RNase A (final concentration of 0.1 μg/μl) for 0.5 h at 37 °C and proteinase K (final concentration of 0.4 μg/μl) for 1 h at 55 °C. DNA was extracted with the QIAquick MinElute PCR Purification Kit (Qiagen, 28,104). Genomic DNA without immunoprecipitation was used as an input control. A sequencing library was prepared using an Illumina Truseq ChIP Sample Prep Kit (Illumina, USA). The pooled libraries were sequenced on an Illumina HiSeq 2000 platform using the 100-bp singled-ended sequencing protocol.

### Strand-specific RNA sequencing (ssRNA-seq)

Total RNA was isolated using TRIzol (Invitrogen, USA) according to the manufacturer’s instructions. RNA quality was assessed with an Agilent 2100 Bioanalyzer. Samples with RNA Integrity Number (RIN) values > 7.5 were selected (Additional file 1: Table S1). Total RNA (1 μg) from the same PFC sample used in ChIP-seq was used to construct the sequencing library following the TruSeq Stranded mRNA Sample Prep (Illumina, USA) protocol. The libraries were pooled and sequenced on an Illumina HiSeq 4000 platform in the 150-bp singled-ended mode.

### Consensus genome and gene annotation construction

To directly compare the histone modification and transcriptome among humans, chimpanzees, and rhesus macaques, a consensus genome and gene annotation for the three species were constructed for further analysis. The consensus genome was constructed as described in [59, 99]. In detail, the pairwise genome alignment files of the human (hg19) and chimpanzee (panTro4) genomes and the human (hg19) and rhesus macaque (rheMac3) genomes, aligned by BLASTZ, were downloaded from the UCSC genome browser (https://genome.ucsc.edu). Based on these alignment files, a multiple genome alignment of three species was constructed using the multi-alignment tool [100]. A human–chimpanzee–macaque consensus genome was further constructed by replacing all discordant sites in the human genome, including mismatches and insertions/deletions, with “N” according to the three-species alignment. The consensus gene annotation was constructed as described previously [56]. Human gene annotation was downloaded from GENCODE (v21; https://www.gencodegenes.org). Chimpanzee and rhesus macaque coordinates based on the genome version panTro4 and rheMac3, respectively, were constructed from human gene annotation using the LiftOver tool (http://www.genome.ucsc.edu/cgi-bin/hgLiftOver). Gene annotation coordinates that had the same exon orders and changed no more than 50% during LiftOver were preserved. The intersection of regions with coordinates mapped on hg19 was subsequently merged and used for further analysis.

### ChIP-seq data preprocessing

The ChIP-seq raw reads were aligned to the consensus genome as described above using bowtie (version 2–2.2.5) with the “--very-sensitive-local” model [101]. Next, duplicated reads were removed using Picard tools (version 1.117; https://broadinstitute.github.io/picard/). Only unique mapped reads were used for peak calling. Statistically genome-wide significant enriched regions with an FDR < 0.01 for H3K4me3 and H3K27ac (termed H3K4me3/H3K27ac raw peaks for short) were identified by comparing the ChIP-seq samples to corresponding input samples using the MACS peak caller (version 1.4.2). The location overlaps of the raw peaks in each of the two samples was estimated by BEDOPS [102]. The raw peaks detected in all three samples within one species were firstly merged as peaks in humans, chimpanzees, and macaques (termed H3K4me3/H3K27ac peaks for short) and then compared among species to divide the peaks into the groups that were shared by three species, two species, and peaks that were unique to one species. Peaks that overlap at their location on the genome were merged for further analysis. The R package ChIPseeker [103] was used to annotate the location of peaks with the closest genes and genomic regions based on the human reference genome (hg19). The region within 3000 bp from known TSSs was defined as the promoter region. The overlap between the H3K4me3/H3K27ac peaks and the promoter region was also estimated by BEDOPS. The read counts on each merged peak were calculated by coverageBed in bedtools [104]. The peak intensities were calculated using read coverage normalized by peak length and the total number of reads mapped to peaks in a sample.

### Strand-specific RNA-seq data preprocessing

RNA-seq raw reads were firstly trimmed with the ea-utils tool (version 1.1.2) [105] and then aligned to the consensus genome constructed as described above with STAR (version 2.4.2) [106] using the default parameters. Unique mapped reads were obtained by removing duplicates with Picard tools (version 1.117) for further analysis. The read counts on each consensus annotation were calculated by coverageBed in bedtools. The gene expression levels were calculated using the read coverage normalized by gene length and the total number of reads mapped to genes in a sample. Only genes with read counts > 0 in at least one species were considered for further analysis.

### Hierarchical clustering and PCA

Pairwise Pearson correlations based on the peak intensity between samples and the proportion of overlapping peaks calculated by BEDOPS as described above were used for hierarchical clustering. Heatmaps were created using the heatmap.2 function from the R package “gplots”. For PCA, the prcomp function in R based on peak intensity was applied with default parameters.

### Identification of species-specific histone peaks and expressed genes

To identify species-specific histone peaks or expressed genes, differential analysis between every two species was performed on ChIP-seq data, RNA-seq data, and ssRNA-seq data using the edgeR v3.36.0, respectively [107]. For each comparison, normalization factors were computed using the calcNormFactors function, which employs the trimmed mean of M-value (TMM) technique; after which, tagwise dispersions were estimated and the read count matrix was subjected to a quasi-likelihood negative binomial generalized log-linear model (glmQLFit) using species as covariates. The resulting *P* values were determined using glmQLFTest. Multiple testing was conducted by applying the Benjamini-Hochberg (BH) method to the *P* values to control the FDR. The average peak coverage or gene expression was calculated for each species, and then the fold change was defined as the ratio of the average value between every two species. Only peaks with FDR < 0.05 and fold change > 1.2 were considered significant. For expressed genes, the fold change threshold was set at 1.5. If a peak or gene showed no significant difference between chimpanzees and macaques but showed a significant difference between humans and the other two primate species, this peak or gene was assigned to the human-specific peak (referred to ^H3K4me3^HP and ^H3K27ac^HP) or gene. Chimpanzee- and macaque-specific peaks or genes were defined by the analogous criteria.

### Functionality enrichment analysis and cell type specificity

Gene Ontology (GO) terms associated with ^H3K4me3^HP- and ^H3K27ac^HP-enriched genes were determined by the Bioconductor package “clusterProfiler” based on hypergeometric distribution [108]. The background gene set was downloaded from the Allen Brain Atlas (https://human.brain-map.org) data portal. GO term categories specific to biological processes with *P* < 0.05 after BH correction were considered to be significantly enriched. Cell type specificity was performed as described previously [99]. For brevity, marker genes subjected to nine major cell types were determined in the mouse brain using single-cell RNA-seq [78]. The cell types contained neuronal subtypes, including S1 pyramidal neurons, CA1 pyramidal neurons, and interneurons, as well as non-neuronal glia cells, including astrocytes, oligodendrocytes, endothelial cells, ependymal cells, mural cells, and microglia. A total of 19,282 human-mouse one-to-one orthologs were downloaded from Ensembl (https://asia.ensembl.org). For each cell type, a hypergeometric test was applied to compare the overrepresentation of marker genes to ^H3K4me3^HP- and ^H3K27ac^HP-enriched genes while using all mouse genes as the background. The BH-corrected *P* < 0.05 was used as the enrichment cutoff.

### PPI network construction

The PPI networks of ^H3K4me3^HP- and ^H3K27ac^HP-enriched genes were constructed using the STRING (Search Tool for the Retrieval of Interacting Genes/Proteins) database (https://cn.string-db.org), which provides a critical assessment and integration of protein–protein interactions, including physical or functional associations [109]. The threshold of the PPI score was set as 0.7 to obtain interactions with higher confidence. As nodes with a higher degree of connectivity make larger contributions to the stability of the network, genes with connectivity degrees > 10 were defined as hub genes using the CentiScaPe plugin [110]. The PPI network was visualized by Cytoscape v3.8.2 (https://cytoscape.org) [111].

### Association between regulatory mechanisms and species-specific genes

Pearson correlation coefficients were calculated between three regulatory mechanisms (TF, H3K4me3, and H3K27ac) and their corresponding target genes specific to the three species. For each regulatory mechanism, the correlation expected by chance was assessed by randomly sampling the same number of non-species-specific genes and their coupled regulators, a procedure that was repeated 100 times.

### Relationship between regulatory mechanisms and human-chimpanzee differences

The association between human-chimpanzee gene expression differences and three regulatory mechanisms was estimated previously [26]. In detail, log_2_ fold changes of gene expression differences and coupled TF expression differences or coupled H3K4me3/H3K27ac coverage differences were fitted with a linear regression model, and variance was inferred using the anova function in R. Only human- and chimpanzee-specific genes detected in the ssRNA-seq dataset were considered. Particularly, the significance of the TFs correlated with corresponding target genes was inspected by the cor.test function in R. TFs with absolute Pearson correlation coefficients > 0.6 and *P* < 0.05 were used in the above analysis. Permutation was performed by randomly coupling genes to those regulators 1000 times to estimate the significance of these associations.

### Putative TFs regulating human-specific genes

TFs showing a significant correlation with their corresponding target genes specific to humans were calculated by checking the absolute Pearson correlation coefficients between the expression profiles of TFs and human-specific target genes detected in the ssRNA-seq dataset. Significance was assessed by comparing the absolute Pearson correlation coefficients calculated within human-specific genes to those calculated between the same TF and its corresponding non-human-specific target genes using a one-sided Wilcoxon rank-sum test. TFs with BH-corrected *P* < 0.05 and absolute Pearson correlation coefficients > 0.6 were determined to be significantly correlated. The expected by chance number of correlated TFs was estimated by shuffling species labels 1000 times for genes.

### Putative human-specific genes regulated by TFs

For each human-specific gene, the gene showing a significant correlation with its corresponding TF was calculated by checking the absolute Pearson correlation coefficients between the expression profile of the human-specific gene and the corresponding TF. To estimate the correlation significance, we randomly chose the same number of genes from all expressed genes as TFs and calculated the absolute Pearson correlation coefficients between the expression profiles of the same gene and randomly chosen TFs. A one-sided Wilcoxon rank-sum test was used to determine the significance of a higher correlation for TFs. Genes with a BH-corrected *P* < 0.05 and absolute Pearson correlation coefficients > 0.6 were inferred to be significantly correlated. The expected by chance number of correlated human-specific genes was assessed by shuffling the species labels 1000 times for TFs.

### Histone-modifying enzymes

Histone-modifying enzymes were retrieved from the HISTome2 database (http://www.actrec.gov.in/histome2/Human/index.php), a knowledgebase of histone proteins, post-translational modifications, and histone-modifying enzymes for multiple organisms with epidrugs [112]. A panel of epigenetic regulators, as well as their targets, were also characterized according to the manually curated EpiFactors database (http://epifactors.autosome.ru), which provides a wide range of information about human proteins and complexes involved in epigenetic regulation [113].

### Association between enzymes and species-specific histone peaks

The Pearson correlation of enzymes derived from the ssRNA-seq dataset and the species-specific H3K4me3 and H3K27ac peaks was calculated using all the expression profiles of all three species. The expected by chance correlation coefficients were further estimated by randomly sampling the same number of non-species-specific histone peaks and repeating the permutation 100 times.

### Putative enzymes mediating species-specific histone peaks

For every enzyme, the correlation between the enzyme and the species-specific histone peaks was compared to the correlation of the same enzyme and the same number of non-species-specific histone peaks using a one-sided Wilcoxon test. Enzymes with *P* < 0.01 were deemed to correlate well with species-specific histone peaks. The average value based on 1000 permutations was defined as the number of significantly correlated enzymes. Furthermore, the expected by chance number of correlated enzymes was calculated by 1000 random samplings of nonenzymatic genes and the same number of non-species-specific histone peaks. It was noteworthy that the test was conducted using all three species together.

## Supplementary Information

**Additional file 1: Table S1. **Sample information. **Table S2.** ChIP-seq mapping statistics. **Table S3.** Normalized read counts of H3K4me3 peaks in human, chimpanzee and macaque PFC used in this study. **Table S4.** Normalized read counts of H3K27ac peaks in human, chimpanzee and macaque PFC used in this study. **Table S5.** List of 1175 H3K4me3 peaks with human-specific gain in PFC. **Table S6.** List of 775 H3K4me3 peaks with human-specific loss in PFC. **Table S7.** Functional enrichment of genes adjacent to ^H3K4me3^HP gain. **Table S8.** Functional enrichment of genes adjacent to ^H3K4me3^HP loss. **Table S9.** List of 483 H3K27ac peaks with human-specific gain in PFC. **Table S10.** List of 316 H3K27ac peaks with human-specific loss in PFC. **Table S11.** Functional enrichment of genes adjacent to ^H3K27ac^HP gain and loss. **Table S12.** Strand-specific RNA-seq mapping statistics. **Table S13.** Raw and normalized read counts in human, chimpanzee and macaque PFC used in this study. **Table S14.** List of 69 TFs and their target genes with absolute Pearson correlation coefficients > 0.6 and *P* values < 0.05. **Table S15.** List of 28 H3K4me3- and two H3K27ac-modifying enzymes.

**Additional file 2: Fig. S1.** Species-specific expressed genes detected in RNA-seq and ssRNA-seq datasets.Overlap of human-, chimpanzee-, and macaque-specific genes detected in RNA-seq and ssRNA-seq.Pearson correlation coefficients of log_2_-transformed fold changes between RNA-seq and ssRNA-seq for each species-specific gene set. All correlation coefficients were calculated using common species-specific expressed genes identified in both datasets. Each symbol represents individual gene, the line shows linear model curves. Different colors represent each pairwise comparison.***P < 0.001. **Fig. S2.** Proportion of type I, II and III genes regulated by species-specific histone peaks. Different colors denote each primate. The dark color denotes H3K4me3 modification, and the light color denotes H3K27ac modification. Significance of overlap between three types and expressed genes regulated by species-specific histone modification is marked by asterisk in each bar. **Fig. S3.** Histone-TF target regulatory network.Association expected by chance between human-chimpanzee gene expression differences and coupled TFs’ expression differences or coupled H3K4me3/H3K27ac coverage differences was done by randomly coupling genes to those regulators 1000 times. Green, TF; orange, H3K4me3; light orange, H3K27ac.Pearson correlation coefficients between log_2_ fold changes of human-chimpanzee expression differences and coupled regulators. The red triangle denotes true value corresponding to each regulatory mechanism.The percentage of gene expression variance between human and chimpanzee explained by coupled regulators.Left bar: number of TFs significantly correlated with human-specific genes detected in ssRNA-seq dataset by comparing the absolute Pearson correlation coefficients calculated within human-specific genes to that calculated between the same TF and its corresponding non-human-specific target genes using one-sided Wilcoxon rank-sum test. The streaked bar represents the average number of correlated TFs expected by chance, estimated by shuffling species’ labels 1000 times for genes. Right bar: number of human-specific genes significantly correlated with their corresponding TFs by comparing the absolute Pearson correlation coefficients between expression profile of human-specific gene and corresponding TFs to that calculated between the same gene and the same number of TFs that randomly selected from all expressed genes using one-sided Wilcoxon rank-sum test. The streaked bar represents the average number of correlated human-specific genes expected by chance, estimated by shuffling species’ labels 1000 times for TFs. **Fig. S4.** Six major brain cell types in Alzheimer’s disease enriched with genes adjacent to ^H3K4me3^HP and ^H3K27ac^HP.The orange represents H3K4me3 and H3K27ac peaks with significant enrichment, green represents H3K4me3 and H3K27ac peaks with significant depletion. For each cell type, a hypergeometric test was applied to compare the overrepresentation of marker genes to ^H3K4me3^HP- and ^H3K27ac^HP-enriched genes while using all of the brain genes as the background. The BH-corrected *P* < 0.05 was used as the enrichment cutoff.

## Data Availability

All data generated or analyzed during this study are included in this published article, its supplementary information files (Additional file 1), and publicly available repositories. Raw data are deposited in the National Omics Data Encyclopedia (NODE; http://www.biosino.org/node) and are available through NODE accession number OEP003561 at https://www.biosino.org/node/project/detail/OEP003561. The ChIP-seq datasets have accession number OEX020247 (https://www.biosino.org/node/experiment/detail/OEX020247), and matched strand-specific RNA-seq datasets have accession number OEX020246 (https://www.biosino.org/node/experiment/detail/OEX020246).

## Abbreviations

ChIP-seq: Chromatin immunoprecipitation sequencing
ssRNA-seq: Strand-specific RNA sequencing
H3K4me3: Histone H3 lysine 4 trimethylation
H3K27ac: Histone H3 lysine 27 acetylation
^H3K4me3^HP: Human-specific H3K4me3 peaks
^H3K27ac^HP: Human-specific H3K27ac peaks
PFC: Prefrontal cortex
TF: Transcription factor
TSS: Transcription start site
TFBS: Transcription factor binding site
FDR: False discovery rate
RIN: RNA Integrity Number
GO: Gene Ontology
AD: Alzheimer’s disease
SZ: Schizophrenia
