# Supplementary figures and images for: Epigenetic regulation of human-specific gene expression in the prefrontal cortex
Source: BMC Biol. 2023 May 24;21:123. doi: 10.1186/s12915-023-01612-3 (PMC10210484; doi:10.1186/s12915-023-01612-3)

**A**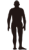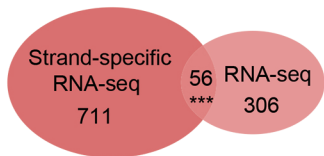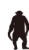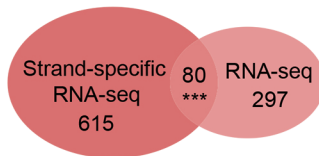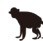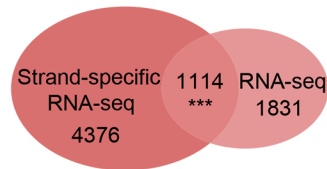**B**

—●— human vs chimp.

—▲— human vs macaque

—\*— chimp. vs macaque

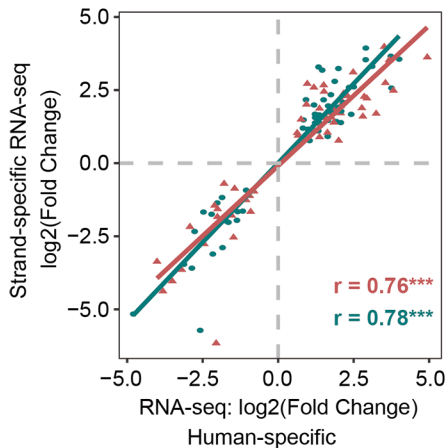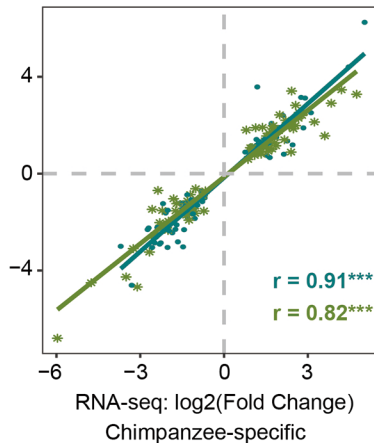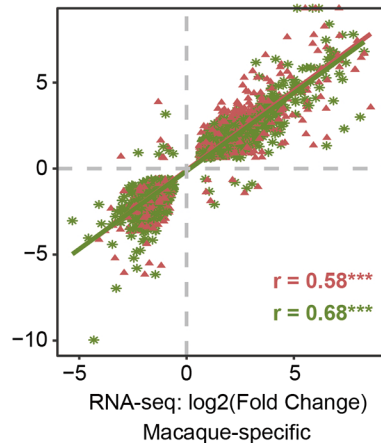

Supplement: Supplementary file 2 — Additional file 2: Fig. S1. Species-specific expressed genes detected in RNA-seq and ssRNA-seq datasets.Overlap of human-, chimpanzee-, and macaque-specific genes detected in RNA-seq and ssRNA-seq.Pearson correlation coefficients of log2-transformed fold changes between RNA-seq and ssRNA-seq for each species-specific gene set. All correlation coefficients were calculated using common species-specific expressed genes identified in both datasets. Each symbol represents individual gene, the line shows linear model curves. Different colors represent each pairwise comparison.***P < 0.001. Fig. S2. Proportion of type I, II and III genes regulated by species-specific histone peaks. Different colors denote each primate. The dark color denotes H3K4me3 modification, and the light color denotes H3K27ac modification. Significance of overlap between three types and expressed genes regulated by species-specific histone modification is marked by asterisk in each bar. Fig. S3. Histone-TF target regulatory network.Association expected by chance between human-chimpanzee gene expression differences and coupled TFs’ expression differences or coupled H3K4me3/H3K27ac coverage differences was done by randomly coupling genes to those regulators 1000 times. Green, TF; orange, H3K4me3; light orange, H3K27ac.Pearson correlation coefficients between log2 fold changes of human-chimpanzee expression differences and coupled regulators. The red triangle denotes true value corresponding to each regulatory mechanism.The percentage of gene expression variance between human and chimpanzee explained by coupled regulators.Left bar: number of TFs significantly correlated with human-specific genes detected in ssRNA-seq dataset by comparing the absolute Pearson correlation coefficients calculated within human-specific genes to that calculated between the same TF and its corresponding non-human-specific target genes using one-sided Wilcoxon rank-sum test. The streaked bar represents the average number of co [file 12915_2023_1612_MOESM2_ESM.zip › Additional file 2/Fig.S1.pdf]

Type I

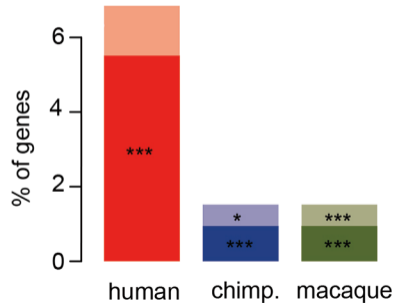

Type II

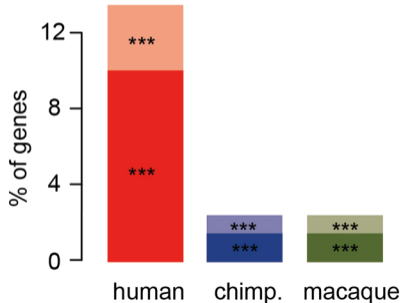

Type III

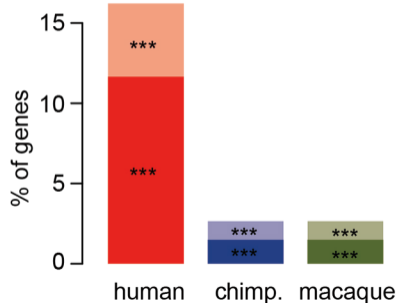

Supplement: Supplementary file 2 — Additional file 2: Fig. S1. Species-specific expressed genes detected in RNA-seq and ssRNA-seq datasets.Overlap of human-, chimpanzee-, and macaque-specific genes detected in RNA-seq and ssRNA-seq.Pearson correlation coefficients of log2-transformed fold changes between RNA-seq and ssRNA-seq for each species-specific gene set. All correlation coefficients were calculated using common species-specific expressed genes identified in both datasets. Each symbol represents individual gene, the line shows linear model curves. Different colors represent each pairwise comparison.***P < 0.001. Fig. S2. Proportion of type I, II and III genes regulated by species-specific histone peaks. Different colors denote each primate. The dark color denotes H3K4me3 modification, and the light color denotes H3K27ac modification. Significance of overlap between three types and expressed genes regulated by species-specific histone modification is marked by asterisk in each bar. Fig. S3. Histone-TF target regulatory network.Association expected by chance between human-chimpanzee gene expression differences and coupled TFs’ expression differences or coupled H3K4me3/H3K27ac coverage differences was done by randomly coupling genes to those regulators 1000 times. Green, TF; orange, H3K4me3; light orange, H3K27ac.Pearson correlation coefficients between log2 fold changes of human-chimpanzee expression differences and coupled regulators. The red triangle denotes true value corresponding to each regulatory mechanism.The percentage of gene expression variance between human and chimpanzee explained by coupled regulators.Left bar: number of TFs significantly correlated with human-specific genes detected in ssRNA-seq dataset by comparing the absolute Pearson correlation coefficients calculated within human-specific genes to that calculated between the same TF and its corresponding non-human-specific target genes using one-sided Wilcoxon rank-sum test. The streaked bar represents the average number of co [file 12915_2023_1612_MOESM2_ESM.zip › Additional file 2/Fig.S2.pdf]

**A**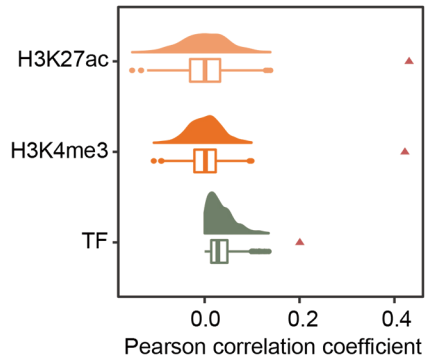**B**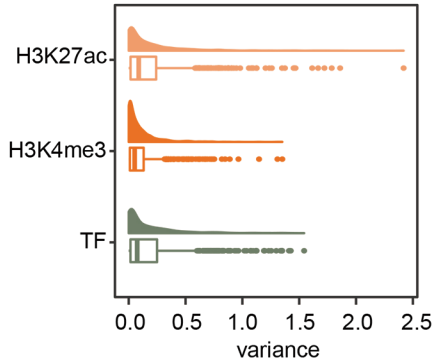**C**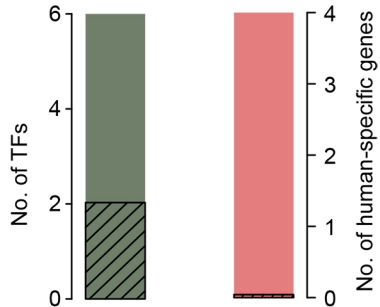

Supplement: Supplementary file 2 — Additional file 2: Fig. S1. Species-specific expressed genes detected in RNA-seq and ssRNA-seq datasets.Overlap of human-, chimpanzee-, and macaque-specific genes detected in RNA-seq and ssRNA-seq.Pearson correlation coefficients of log2-transformed fold changes between RNA-seq and ssRNA-seq for each species-specific gene set. All correlation coefficients were calculated using common species-specific expressed genes identified in both datasets. Each symbol represents individual gene, the line shows linear model curves. Different colors represent each pairwise comparison.***P < 0.001. Fig. S2. Proportion of type I, II and III genes regulated by species-specific histone peaks. Different colors denote each primate. The dark color denotes H3K4me3 modification, and the light color denotes H3K27ac modification. Significance of overlap between three types and expressed genes regulated by species-specific histone modification is marked by asterisk in each bar. Fig. S3. Histone-TF target regulatory network.Association expected by chance between human-chimpanzee gene expression differences and coupled TFs’ expression differences or coupled H3K4me3/H3K27ac coverage differences was done by randomly coupling genes to those regulators 1000 times. Green, TF; orange, H3K4me3; light orange, H3K27ac.Pearson correlation coefficients between log2 fold changes of human-chimpanzee expression differences and coupled regulators. The red triangle denotes true value corresponding to each regulatory mechanism.The percentage of gene expression variance between human and chimpanzee explained by coupled regulators.Left bar: number of TFs significantly correlated with human-specific genes detected in ssRNA-seq dataset by comparing the absolute Pearson correlation coefficients calculated within human-specific genes to that calculated between the same TF and its corresponding non-human-specific target genes using one-sided Wilcoxon rank-sum test. The streaked bar represents the average number of co [file 12915_2023_1612_MOESM2_ESM.zip › Additional file 2/Fig.S3.pdf]

**A**

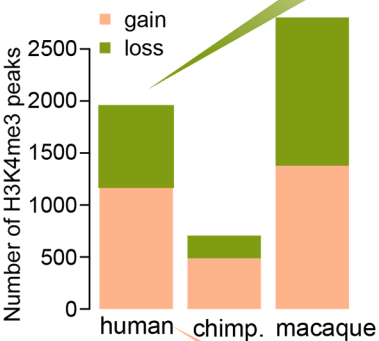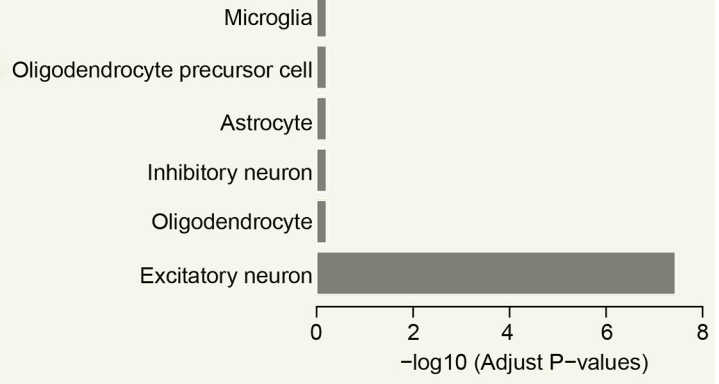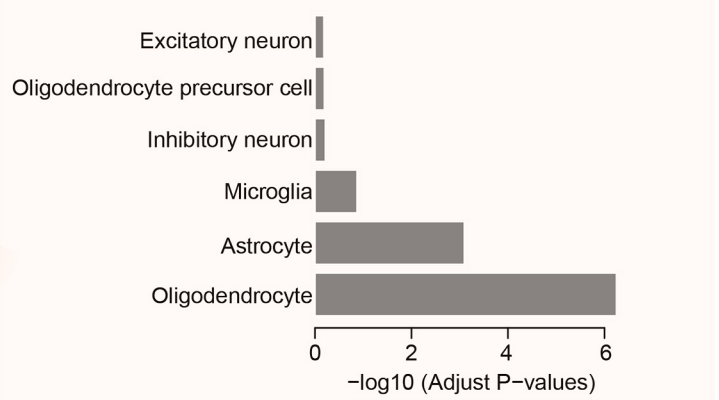

**B**

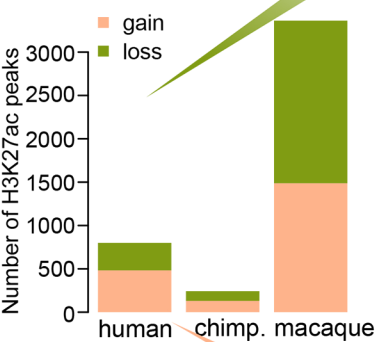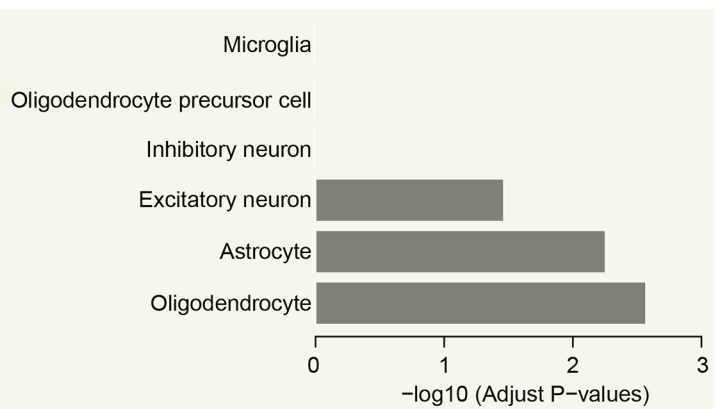

Supplement: Supplementary file 2 — Additional file 2: Fig. S1. Species-specific expressed genes detected in RNA-seq and ssRNA-seq datasets.Overlap of human-, chimpanzee-, and macaque-specific genes detected in RNA-seq and ssRNA-seq.Pearson correlation coefficients of log2-transformed fold changes between RNA-seq and ssRNA-seq for each species-specific gene set. All correlation coefficients were calculated using common species-specific expressed genes identified in both datasets. Each symbol represents individual gene, the line shows linear model curves. Different colors represent each pairwise comparison.***P < 0.001. Fig. S2. Proportion of type I, II and III genes regulated by species-specific histone peaks. Different colors denote each primate. The dark color denotes H3K4me3 modification, and the light color denotes H3K27ac modification. Significance of overlap between three types and expressed genes regulated by species-specific histone modification is marked by asterisk in each bar. Fig. S3. Histone-TF target regulatory network.Association expected by chance between human-chimpanzee gene expression differences and coupled TFs’ expression differences or coupled H3K4me3/H3K27ac coverage differences was done by randomly coupling genes to those regulators 1000 times. Green, TF; orange, H3K4me3; light orange, H3K27ac.Pearson correlation coefficients between log2 fold changes of human-chimpanzee expression differences and coupled regulators. The red triangle denotes true value corresponding to each regulatory mechanism.The percentage of gene expression variance between human and chimpanzee explained by coupled regulators.Left bar: number of TFs significantly correlated with human-specific genes detected in ssRNA-seq dataset by comparing the absolute Pearson correlation coefficients calculated within human-specific genes to that calculated between the same TF and its corresponding non-human-specific target genes using one-sided Wilcoxon rank-sum test. The streaked bar represents the average number of co [file 12915_2023_1612_MOESM2_ESM.zip › Additional file 2/Fig.S4.pdf]
